# Correlates of Receiving Guideline-Concordant Postpartum Health Services in the Community Health Center Setting

**DOI:** 10.1089/whr.2021.0084

**Published:** 2022-02-07

**Authors:** Kathryn Wouk, Alan C. Kinlaw, Narges Farahi, Henry Pfeifer, Brandon Yeatts, Moo Kho Paw, Whitney R. Robinson

**Affiliations:** ^1^ Department of Maternal and Child Health, Carolina Global Breastfeeding Institute, Gillings School of Global Public Health, The University of North Carolina at Chapel Hill, North Carolina, USA.; ^2^ Division of Pharmaceutical Outcomes and Policy, University of North Carolina School of Pharmacy, Chapel Hill, North Carolina, USA.; ^3^ Cecil G. Sheps Center for Health Services Research, University of North Carolina at Chapel Hill, North Carolina, USA.; ^4^ Department of Family Medicine, The University of North Carolina at Chapel Hill, Chapel Hill, North Carolina, USA.; ^5^ Piedmont Health Services, Chapel Hill, North Carolina, USA.; ^6^ Department of Physician Assistant Studies, East Carolina University, Greenville, North Carolina, USA.; ^7^ School of Medicine, Virginia Commonwealth University, Richmond, Virginia, USA.; ^8^ Department of Epidemiology, Gillings School of Global Public Health, The University of North Carolina at Chapel Hill, Chapel Hill, North Carolina, USA.

**Keywords:** health care utilization, HER, marginalized populations, federally qualified health centers, postpartum

## Abstract

***Introduction:*** New clinical guidelines recommend comprehensive and timely postpartum services across 3 months after birth. Research is needed to characterize correlates of receiving guideline-concordant, quality postpartum care in federally qualified health centers serving marginalized populations.

***Methods:*** We abstracted electronic health record data from patients who received prenatal health care at three health centers in North Carolina to characterize quality postpartum care practices and to identify correlates of receiving quality care. We used multivariable log-binomial regression to estimate associations between patient, provider, and health center characteristics and two quality postpartum care outcomes: (1) timely care, defined as an initial assessment within the first 3 weeks and at least one additional visit within the first 3 months postpartum; and (2) comprehensive care, defined as receipt of services addressing family planning, infant feeding, chronic health, mood, and physical recovery across the first 3 months.

***Results:*** In a cohort of 253 patients, 60.5% received comprehensive postpartum care and 30.8% received timely care. Several prenatal factors (adequate care use, an engaged patient–provider relationship) and postpartum factors (early appointment scheduling, exclusive breastfeeding, and use of enabling services) were associated with timely postpartum care. The most important correlate of comprehensive services was having more than one postpartum visit during the first 3 months postpartum.

***Discussion:*** Identifying best practices for quality postpartum care in the health center setting can inform strategies to reduce health inequities. Future research should engage community stakeholders to define patient-centered measures of quality postpartum care and to identify community-centered ways of delivering this care.

## Introduction

Maternal mortality and morbidity are increasing in the United States,^1–4^ with stark disparities by race, ethnicity, geography, and place of birth.^5–8^ Black and Indigenous people have the highest risk of maternal mortality and morbidity.^2,5,7,9^ In addition, Hispanic and Asian/Pacific Islander populations experience higher risks of severe maternal morbidity compared with non-Hispanic white populations.^10–12^ Low income, uninsured, and rural populations, especially those in the United States South, all have elevated rates of severe maternal morbidity.^7,8^

The postpartum period is a critical window for delivering quality health care services to reduce these disparities in maternal mortality and morbidity.^13,14^ Over half of all pregnancy-related deaths occur in the postpartum period.^2^ One-third of these deaths occur after the first week after birth when health care is typically irregular, fractured, and misaligned with women's health priorities.^15,16^ In addition, severe maternal morbidity in the postpartum period has been steadily increasing over the past 20 years.^3,17^

As one strategy to reduce mortality and morbidity in the postpartum period, the American College of Obstetricians and Gynecologists (ACOG) recently revised their clinical guidelines to recommend shifting from a single visit at 6 weeks postpartum to an ongoing process of person-centered health care delivered over a longer postpartum period of 3 months.^18,19^ These clinical recommendations do not include an explicit definition of quality postpartum care, but they encourage providers to holistically address each new parent's health needs as they arise, with both timely and comprehensive services.

Federally qualified health centers (referred to throughout as community health centers) offer primary care and enabling services across the prenatal and postpartum periods regardless of insurance coverage or ability to pay, reaching approximately one-fifth of all low-income birthing people in the United States.^20^ People of color and low-income populations at highest risk for maternal mortality and morbidity disproportionately receive outpatient perinatal health services at community health centers as they are least likely to have perinatal insurance coverage.^21^

This issue is particularly salient in states such as North Carolina, which have not expanded Medicaid and do not extend health insurance to all pregnant people regardless of citizenship status.^2^ Community health centers provide safety net services for especially large proportions of low-income and immigrant families, and their patients are more likely to return for postpartum care compared with general populations.^22^

Identifying best practices for guideline-concordant, quality postpartum care in the community health center setting can help inform strategies to reduce maternal health inequities. Patients cared for in community health centers have been found to have fewer racial and ethnic disparities in birth outcomes compared with the general U.S. population.^20^ Studies have documented lower quality maternity care at hospitals serving Black, Latina, and rural populations, proposing that this lower quality care may drive maternal health inequities.^23–27^ However, the quality of postpartum care in community health centers has not yet been investigated, as most research focused on improving attendance at the single postpartum visit^22,28–30^ before the 2018 call for a paradigm shift in “fourth trimester” care.^18,31^

To contribute to the nascent literature on quality postpartum care since the change in clinical guidance, we conducted an exploratory analysis to understand current postpartum care practices in the community health center setting. We used electronic health record (EHR) data from patients who received prenatal health care at three community health centers in North Carolina to characterize guideline-concordant, quality postpartum care and to identify correlates of receiving this quality postpartum care. We examined associations between health center, provider, and patient characteristics and quality postpartum care informed by the recent ACOG guidelines.

## Materials and Methods

### Study population

We used EHR data from perinatal patient encounters of people who received prenatal care at one of three outpatient community health centers who gave birth or experienced a miscarriage or stillbirth in 2018. The three community health centers belong to a network of community health centers serving 14 rural and urban counties across central North Carolina. They served nearly 50,000 patients in 2018, and 75% of these patients were of non-white race or of Hispanic/Latino ethnicity, compared with 29.4% of North Carolina residents.^32^ Approximately 86% of patients at these centers had a household income at or below 100% of the poverty line and nearly half were uninsured.

This exploratory study was conducted in collaboration with three facilities in this community health center network whose diverse site characteristics (varying rural and urban locations, patient population demographics, and staff size) and provider teams (comprising family physicians and residents, nurse practitioners, physician assistants, and/or internal medicine physicians) facilitated data abstraction from a single EHR system to generate findings that will be relevant across different contexts. These facilities provide outpatient prenatal and postpartum care and coordinate intrapartum care with local hospitals where their patients deliver.

### Data source

We drew from our prior systematic review^33^ of patient-level, provider-level, and health center-level predictors of postpartum care use in marginalized populations to enumerate potential variables associated with receiving guideline-concordant quality postpartum care.

Targeting these key variables, we abstracted discrete data and free text on patient encounters from the GE Centricity EHR software into a Research Electronic Data Capture (REDCap) structured database. REDCap is a web application for building and managing online surveys and databases,^34^ which is secure and compliant with the Health Insurance Portability and Accountability Act (HIPAA). The lead researcher (K.W.) created separate REDCap data collection forms for storing demographic data as well as encounter data from prenatal visit notes, intrapartum hospital notes scanned into the EHR, and postpartum visit notes.

For each patient who received prenatal care at one of the three health centers and gave birth or experienced a miscarriage or stillbirth in 2018, we abstracted data from all visits during their pregnancy through 12 weeks postpartum. Data abstraction involved mostly free text review owing to the lack of discrete fields for many of the variables of interest and the lack of a standardized template in the EHR for documentation of the postpartum visit. Data collection forms were edited to incorporate input from health care providers and information technology administrators at the community health network, and appropriate variable construction was confirmed with technical support from the North Carolina Translational and Clinical Sciences Institute.

The lead researcher (K.W.) trained a team of medical scribes (H.F., B.Y., and M.P.) employed at the community health centers to abstract data into the structured REDCap database. Training involved K.W. double-coding 20 charts entered by each abstractor to review and discuss discrepancies to improve agreement before proceeding. Record audits were also carried out throughout the data abstraction process of approximately four records per data abstractor each month to verify data quality over time. This study was approved by the Institutional Review Board at the University of North Carolina at Chapel Hill (IRB #19-0423) and by the board of the community health center network.

### Outcomes

Based on the 2018 ACOG recommendations,^18^ we classified patients as receiving quality postpartum care based on separate dichotomous variables for timeliness and comprehensiveness of care. First, we classified each patient as receiving timely postpartum care if they had both an initial assessment within the first 3 weeks postpartum and at least one additional visit within the first 3 months postpartum. Second, we classified each patient as receiving comprehensive postpartum care based on ACOG's suggested components of a postpartum care plan,^35^ as long as each of the five components were documented at any visit within the first 3 months postpartum:

(1) sexuality, contraception, and birth spacing (family planning counseling and/or contraceptive provision, including any contraceptive method started in the hospital immediately postpartum); (2) discussion of infant feeding status and/or offer of infant feeding support, (3) chronic disease management for patients with a documented chronic health issue at the first prenatal visit (including a tobacco use screen among patients who reported current use or a history of tobacco use); (4) mood (mental health screen via Edinburgh Postnatal Depression Scale or Patient Health Questionnaire); and (5) physical recovery from birth and/or pregnancy-related health issues, including a blood pressure check in the first 10 days among patients with hypertensive disorders (Table 1).

**Table 1.** Quality Postpartum Care Outcomes

Outcome	Components	Details
Timely postpartum care	Initial assessment in the first 3 weeks At least one additional visit in the first 3 months	Initial assessment was via phone or in person with a health care provider The additional visit was in person
Comprehensive postpartum services	Sexuality, contraception, and birth spacing	Counseling on sexuality, birth spacing, and/or contraceptive options Contraceptive provision
	Infant feeding	Provider documents infant feeding information Support, education, resources, or problem-solving offered by the provider or a lactation consultant
	Chronic disease management	Chronic hypertension or history of gestational hypertension Diabetes mellitus or history of gestational diabetes Thyroid disorder Anemia Hyperlipidemia Tobacco use screen where patient smokes or has a history of smoking Mood or anxiety disorder
	Mood	Edinburgh Postnatal Depression Scale screen Patient Health Questionnaire-9/2 screen
	Physical recovery from birth and/or pregnancy-related health issues	Blood pressure check in the first 10 days among those with hypertensive disorders Counseling on physical activity and nutrition for weight management Pain, incontinence Glucose screening among those with gestational diabetes

### Correlates of guideline-concordant postpartum care

Patient characteristics extracted from the EHR included parity, maternal age, insurance status, chronic diseases (hypertensive disorders, diabetes mellitus or a history of gestational diabetes, thyroid disorders, anemia, hyperlipidemia, tobacco use, or prepregnancy mood or anxiety disorder present at the first prenatal visit), medical complications beginning during pregnancy (gestational diabetes, hypertensive disorders of pregnancy, anemia, perinatal mood or anxiety disorder), delivery type, and a dichotomous variable for exclusive breastfeeding status at the first postpartum visit (exclusive vs. any/none).

We also used the total number of prenatal visits and the timing of the first prenatal visit to construct a dichotomous variable for the adequacy of prenatal care utilization through the Adequacy of Prenatal Care Utilization Index to capture both adequacy of initiation and of received services once care has begun^36^ (Supplementary Table S1).

Provider characteristics included type of health care provider at the patient's first prenatal visit at the community health center (Family Medicine Physician, Family Nurse Practitioner, Internal Medicine Physician, or Physician Assistant) and the consistency of the health care provider (defined as the number of unique providers seen across pregnancy). Based on patient data and a Qualtrics survey sent to all the health care providers identified in our data set (response rate 36/36, with 5 reported by the medical director because the provider no longer worked at the center), we classified whether the patient and provider had concordant race/ethnicity and whether or not the provider was able to communicate in the patient's preferred language.

Health center characteristics included an identifier of the facility where a patient's first prenatal visit in the community health center network occurred to control for site-level differences: two sites are located in urban counties and one in a rural county; the number of staff ranges from 45 to 85 across the sites; the number and type of provider team differs by site, and only one site has resident family physicians who have hospital privileges and attend some deliveries. Additional health center characteristics included the time since delivery at which the postpartum visit was scheduled and any enabling services used by the patient (the Special Supplemental Nutrition Program for women, infants, and children [WIC], nutrition, dental, behavioral health, and care management).

### Statistical analysis

We present the associations between prespecified variables and the two quality postpartum care outcomes of interest. We used multivariable log-binomial regression to estimate risk ratios (RRs) for prenatal variables and prevalence ratios for postpartum variables, because postpartum characteristics were ascertained cross-sectionally with outcomes at the postpartum visit.^37^ We chose this modeling framework instead of logistic regression because odds ratios would overestimate risk/prevalence ratios of primary interest, and difficulties translating odds ratios from logistic regression into risk or prevalence ratio estimates.^37^

We conducted separate log-binomial regression analyses for each outcome measure (*i.e.*, timely and comprehensive postpartum care). We present crude and adjusted estimates of association along with 95% confidence intervals (CIs) to assess precision. To avoid the Table 2 fallacy for adjusted estimates,^38^ we constructed one directed acyclic graph^39,40^ (Supplementary Fig. S1) for the conceptual model for each outcome, and estimated each variable's association with the outcome, adjusting only for the minimal sufficient set of covariates specific to that exposure-outcome dyad (Supplementary Table S1). Statistical analyses were performed using SAS 9.4 (SAS Institute, Cary, NC), and DAGitty software^41^ was used to enumerate covariate adjustment sets.

**Table 2.** Descriptive Characteristics

	Overall (*N* = 253)	
Variable	*n*	%
Patient-level characteristics		
Race/ethnicity group^a^		
Hispanic/Latina/white	173	68.4)
Hispanic/Latina/Black	12	4.7)
Hispanic/Latina/Indian	1	0.4)
Asian	25	9.9)
Non-Hispanic Black	28	11.1)
Non-Hispanic white	14	5.5)
Preferred language		
Spanish	144	56.9
English	88	34.8
Karen/Burmese/Chin Falam	15	5.9
Other^b^	6	2.4
Insurance status^c^		
No insurance	143	56.5
Medicaid	82	32.4
Private	28	11.1
Maternal age		
Teen (<20 years)	22	8.7
20–35 years	163	64.4
Advanced maternal age (35 and older)	68	26.9
Marital status		
Married/partnered	183	73.2
Single/divorced	67	26.8
Missing	3	
Parity		
Nulliparous	51	20.2
Multiparous	202	79.8
Adequacy of prenatal care^d^		
Adequate plus	72	28.5
Adequate	102	40.3
Intermediate	64	25.3
Inadequate	15	5.9
Medical complications during pregnancy^e^	91	36.0
Gestational diabetes	21	8.3
Hypertensive disorder of pregnancy	20	7.9
Anemia	42	16.6
Perinatal mood or anxiety disorder	23	9.1
Chronic health issue present at first prenatal visit	62	24.5
Chronic hypertension or history of gestational hypertension	17	6.7
Diabetes mellitus or history of gestational diabetes	21	8.3
Thyroid disorder	11	4.3
Anemia	12	4.7
Hyperlipidemia	3	1.2
Tobacco use	13	5.1
Mood or anxiety disorder	49	19.4
Birth outcome		
Live birth	245	96.8
Live birth with neonatal demise	3	1.2
Miscarriage	4	1.6
Stillbirth	1	0.4
Delivery type		
Vaginal	192	75.9
Cesarean	57	22.5
Miscarriage	4	1.6
Exclusive breastfeeding at first postpartum visit		
Yes	82	39.6
No	125	60.4
Provider-level characteristics		
Type of health care provider^f^		
Family medicine physician	133	52.6
Family nurse practitioner	98	38.7
Physician assistant	20	7.9
Internal medicine physician	2	0.8
Provider speaks patient's preferred language^f^		
Yes	232	91.7
No	21	8.3
Racial/ethnic concordance of patient/provider^f^		
Yes	31	12.3
No	222	87.8
Number of health care providers seen in pregnancy		
1	109	43.1
2–8	144	56.9
Health center-level characteristics		
Received enabling services in pregnancy^g^		
Yes	149	58.9
No	104	41.1
Postpartum visit scheduled in the first week postpartum		
Yes	58	63.7
No	33	36.3
Missing	162	
Received enabling services at first postpartum visit^g^		
Yes	78	30.8
No	175	69.2

^a^ These terms reflect the discrete racial and ethnic group categories documented in the electronic health record.

^b^ Includes Arabic (*n* = 2), Kinyarwanda (*n* = 1), Mandarin (*n* = 1), Swahili (*n* = 1), Somali (*n* = 1).

^c^ Insurance status was documented at the first prenatal visit.

^d^ The adequacy of prenatal care utilization (APNCU) index classifies prenatal care use according to the adequacy of initiation and of services received according to guidelines (one visit per month through 28 weeks, one visit every 2 weeks through 36 weeks, and one visit per week after, adjusted for the timing of initiation).

^e^ Medical complications during pregnancy are new conditions that arise after the first prenatal visit.

^f^ Defined for the patient's first prenatal visit provider, because this provider often continued to care for the patient across pregnancy.

^g^ Enabling services included WIC, nutrition, dental, behavioral health, and/or care management.

WIC, women, infants, and children.

## Results

### Descriptive characteristics

We identified 286 patients with a birth, miscarriage, or stillbirth in 2018 who received prenatal care at one of the three clinics in our cohort. Among these 286 patients, 33 were excluded based on predelivery information: 25 patients transferred care outside of the community health center network, 7 relocated, and 1 was incarcerated. Our analytic cohort included the remaining 253 patients—248 of whom gave live birth (1 with a neonatal demise), 4 with miscarriage, and 1 with stillbirth. Descriptive characteristics of the cohort up to the birth event are given in Table 2.

Nearly 74% of the cohort was Hispanic/Latina, 11% non-Hispanic Black, almost 10% Asian (mostly Burmese immigrants), and ∼5% non-Hispanic white. Most of the sample were married or partnered (72%) and multiparous (80%). Most patients received adequate prenatal care or better (69%). Baseline prevalence of chronic health issues was 25%, with mood or anxiety disorder being most common (19%). Among the 36% of patients who experienced a medical complication during pregnancy, anemia was most common (17%). Vaginal delivery occurred for 76% of patients in the cohort.

Most patients saw a family medicine physician (53%) or a family nurse practitioner (39%) at their first prenatal visit, and 43% of patients only saw this one provider for all their prenatal care. Only 12% of the sample were racially and ethnically concordant with their first prenatal health care provider; however, this health care provider almost always (92%) spoke the patient's preferred language.

At the health center level, ∼60% of patients received enabling services at some point across their pregnancy, and almost one-third received these services at their first postpartum visit. Data on the time since delivery at which the postpartum visit was scheduled were only available for 91 patients, and ∼64% of this sample had a postpartum visit scheduled in the first week.

### Guideline-concordant postpartum care characteristics

Of the 253 patients in our sample, 89% returned for at least one postpartum visit in the first 3 months and 42% had an initial contact with the health care provider in the first 3 weeks. Comprehensive postpartum care was more prevalent than timely care, and most patients were asked about their infant feeding status and/or offered support; counseled on family planning and/or offered a contraceptive; screened for a mood or anxiety disorder; and asked about birth or pregnancy-related health issues.

**FIG. 1.**
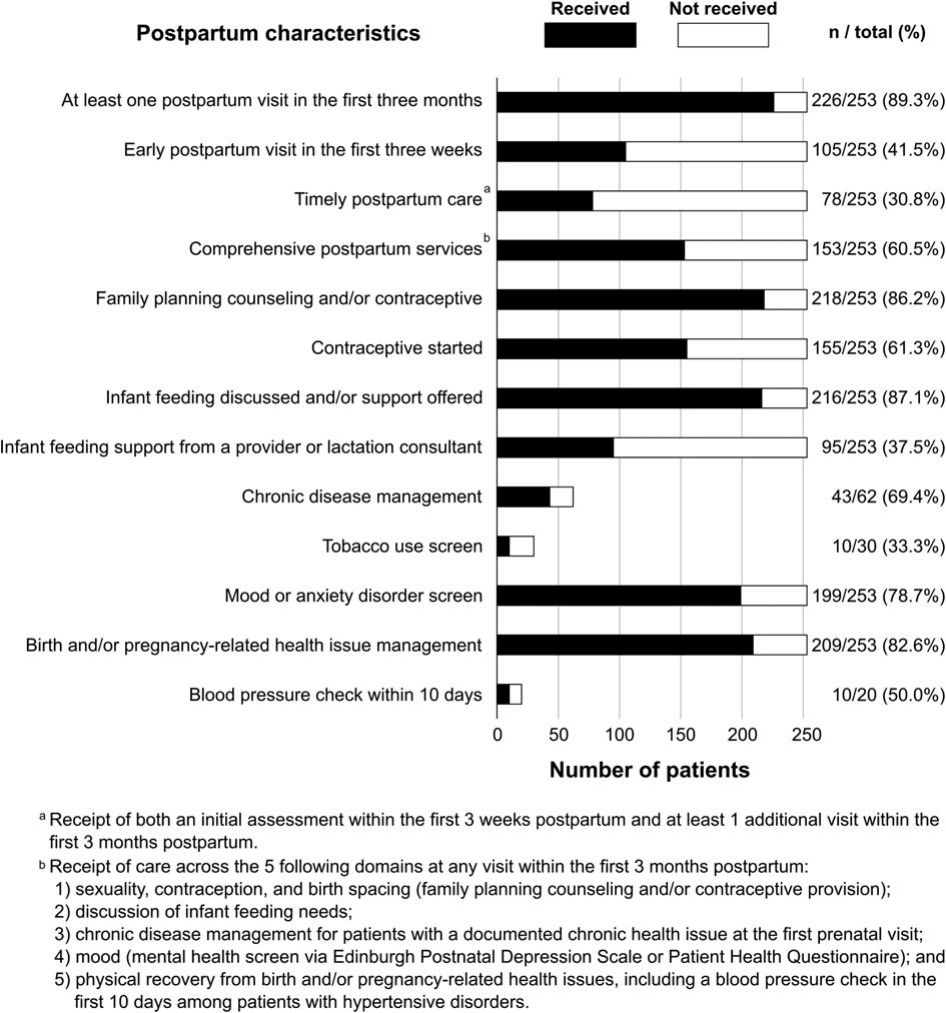
Postpartum characteristics.

Fewer patients received support for infant feeding (whether breast or formula feeding) from the provider or a lactation consultant; a blood pressure check within 10 days; or a tobacco use screen (Fig. 1). Almost 23% of the sample received both timely postpartum care and comprehensive services, whereas nearly one-third received neither timely care nor comprehensive services (Supplementary Table S2).

### Timely postpartum care outcome

Approximately one-third of the sample (31%) received quality postpartum care as defined by timeliness. Patients who had received adequate or “adequate-plus” levels of prenatal care engagement or had a documented mood or anxiety disorder during pregnancy had increased likelihood of receiving timely postpartum care (Table 3). Patients who received prenatal care from a family nurse practitioner more frequently received timely postpartum care compared with patients who received care from a family medicine physician (adjusted RR: 2.1, 95% CI: 1.2–3.7).

**Table 3.** Estimates of Association Between Patient, Provider, and Health Center Characteristics and Receipt of Timely Postpartum Care

Characteristic	*n*/*N*^a^	Crude risk (%)	Crude RR (95% CI)^b^	Adjusted risk (%)	Adjusted RR (95% CI)
Adequacy of prenatal care^c^					
Inadequate/intermediate	16/79	20	(ref.)	15	(ref.)
Adequate/adequate plus	62/174	36	1.8 (1.1–2.8)	26	1.8 (1.1–2.8)
Medical issue during pregnancy^d^					
No	47/162	29	(ref.)	36	(ref.)
Yes	31/91	34	1.1 (0.7–1.6)	38	1.0 (0.7–1.5)
Mood or anxiety disorder during pregnancy					
No	54/197	27	(ref.)	31	(ref.)
Yes	24/56	43	1.6 (1.1–2.3)	47	1.5 (1.1–2.2)
Parity					
Multiparous	60/202	30	(ref.)	31	(ref.)
Primiparous	18/51	35	1.2 (0.8–1.8)	38	1.2 (0.8–2.0)
Insurance status					
Medicaid/private insurance	38/110	35	(ref.)	—	—
Uninsured	40/143	28	0.8 (0.6–1.2)	—	—
Maternal age (years)					
<20	8/22	36	(ref.)	—	—
20–34	46/163	28	0.8 (0.4–1.4)	—	—
35 and older	24/68	35	1.0 (0.5–1.8)	—	—
Number of providers seen across pregnancy					
1 provider	29/109	27	(ref.)	16	(ref.)
>1 provider	49/144	34	1.3 (0.9–1.9)	22	1.3 (0.9–2.0)
Type of prenatal provider^e^					
Family medicine physician	34/133	26	(ref.)	32	(ref.)
Family nurse practitioner	38/98	39	1.5 (1.1–2.2)	68	2.1 (1.2–3.7)
Physician assistant/internal medicine physician	6/22	27	1.1 (0.5–2.2)	30	0.9 (0.4–2.0)
Racial/ethnic concordance of patient/provider^e^					
No	70/222	32	(ref.)	33	(ref.)
Yes	8/31	26	0.8 (0.4–1.5)	27	0.8 (0.4–1.5)
Received enabling services in pregnancy^f^					
No	33/104	32	(ref.)	25	(ref.)
Yes	45/149	30	1.0 (0.7–1.4)	22	0.9 (0.6–1.3)
Delivery type					
Cesarean	19/57	33	(ref.)	43	(ref.)
Vaginal	58/192	30	0.9 (0.6–1.4)	40	0.9 (0.6–1.4)
Postpartum visit scheduled in the first week postpartum					
No	2/33	6	(ref.)	5	(ref.)
Yes	21/55	38	6.3 (1.6–25.2)	32	6.5 (1.6–26.3)
Exclusive breastfeeding at first postpartum visit^g^					
No	32/125	26	(ref.)	16	(ref.)
Yes	37/82	45	1.8 (1.2–2.6)	29	1.8 (1.2–2.6)
Received enabling services at first postpartum visit^f^^,^^g^					
No	43/175	25	(ref.)	33	(ref.)
Yes	35/78	45	1.8 (1.3–2.6)	62	1.9 (1.3–2.7)

Timely care defined as an initial assessment in the first 3 weeks followed by a comprehensive visit in the first 3 months after birth.

^a^ (*n*/*N*): *n* = Number of patients experiencing the outcome divided by *N* = number of patients classified by each level of the characteristic.

^b^ Log-binomial regression was used to estimate RRs.

^c^ Defined as inadequate/intermediate versus adequate/adequate plus using the Kotelchuck index.

^d^ Medical complications of pregnancy include gestational diabetes/diabetes mellitus, hypertensive disorders, anemia, or thyroid disorders.

^e^ Defined for the patient's first prenatal visit provider, because this provider often continued to care for the patient across pregnancy.

^f^ Enabling services included WIC, nutrition, dental, behavioral health, and/or care management.

^g^ Prevalence ratio reported due to cross-sectional exposure and outcome assessment.

CI, confidence interval; RR, risk ratio.

The racial/ethnic concordance of patient and provider were associated with this outcome. Timely care was more common for patients who saw more than one health care provider across pregnancy (adjusted RR: 1.3, 95% CI: 0.9–1.9). Having a postpartum visit scheduled in the first week postpartum versus after the first week was associated with more timely postpartum care, although data on scheduling was only available for a small proportion of the sample (*N* = 91). Exclusive breastfeeding and receiving enabling services at the time of the first postpartum visit were both associated with a higher prevalence of timely postpartum care.

### Comprehensive services outcome

Three-fifths (61%) of the sample received quality postpartum care defined as receiving all recommended services across any number of visits in the 3 months postpartum. Whereas attending an early postpartum visit in the first 3 weeks was not associated with receiving comprehensive postpartum services, attending a higher number of postpartum visits was associated with a higher prevalence of receiving comprehensive services that addressed all recommended health domains (Table 4). Receiving at least adequate levels of prenatal care was associated with a 20% relative increase in receipt of comprehensive services, although the CI for this estimate included the null value (adjusted RR: 2.1, 95% CI: 1.2–3.7).

**Table 4.** Estimates of Association Between Patient, Provider, and Health Center Characteristics and Receipt of Comprehensive Services

Characteristic	*n*/*N*^a^	Crude risk (%)	Crude RR (95% CI)^b^	Adjusted risk (%)	Adjusted RR (95% CI)
Adequacy of prenatal care^c^					
Inadequate/intermediate	45/79	57	(ref.)	55	(ref.)
Adequate/adequate plus	108/174	62	1.1 (0.9–1.4)	64	1.2 (0.9–1.5)
Medical issue during pregnancy^d^					
No	102/162	63	(ref.)	52	(ref.)
Yes	51/91	56	0.9 (0.7–1.1)	45	0.9 (0.7–1.1)
Mood or anxiety disorder during pregnancy					
No	117/197	59	(ref.)	46	(ref.)
Yes	36/56	64	1.1 (0.9–1.4)	53	1.1 (0.9–1.4)
Parity					
Multiparous	124/202	61	(ref.)	49	(ref.)
Primiparous	29/51	57	0.9 (0.7–1.2)	50	1.0 (0.8–1.4)
Insurance status					
Medicaid/private insurance	62/110	56	(ref.)	**—**	**—**
Uninsured	91/143	64	1.1 (0.9–1.4)	**—**	—
Maternal age (years)					
<20	11/22	50	(ref.)	**—**	**—**
20–34	98/163	60	1.2 (0.8–1.9)	**—**	**—**
35 and older	44/68	65	1.3 (0.8–2.0)	—	—
Number of providers seen across pregnancy					
1 provider	64/109	59	(ref.)	52	(ref.)
>1 provider	89/144	62	1.1 (0.9–1.3)	55	1.0 (0.8–1.3)
Type of prenatal provider^e^					
Family medicine physician	77/133	58	(ref.)	54	(ref.)
Nurse practitioner/family practice nurse	65/98	66	1.1 (0.9–1.4**)**	59	1.1 (0.9–1.4)
Physician assistant/internal medicine physician	11/22	50	0.9 (0.6–1.3)	47	0.9 (0.6–1.4)
Racial/ethnic concordance of patient/provider^e^					
No	136/222	61	(ref.)	53	(ref.)
Yes	17/31	55	0.9 (0.6–1.3)	51	1.0 (0.7–1.4)
Received enabling services in pregnancy^f^					
No	66/104	63	(ref.)	64	(ref.)
Yes	87/149	58	0.9 (0.8–1.1)	56	0.9 (0.7–1.1)
Delivery type					
Cesarean	36/57	63	(ref.)	73	(ref.)
Vaginal	117/192	61	1.0 (0.8–1.2)	59	0.8 (0.6–1.0)
Postpartum visit scheduled in the first week postpartum					
No	18/33	55	(ref.)	41	(ref.)
Yes	26/55	47	0.9 (0.6–1.3)	38	0.9 (0.6–1.4)
Exclusive breastfeeding at first postpartum visit^g^					
No	93/125	74	(ref.)	63	(ref.)
Yes	52/82	63	0.9 (0.7–1.0)	54	0.9 (0.7–1.0)
Received enabling services at first postpartum visit^f^^,^^g^					
No	102/175	58	(ref.)	74	(ref.)
Yes	51/78	65	1.1 (0.9–1.4)	86	1.2 (1.0–1.4)
Number of postpartum visits in the first 3 months^g^					
0–1	60/125	48	(ref.)	49	(ref.)
More than 1	93/128	73	1.5 (1.2–1.9)	76	1.6 (1.2–2.0)
Postpartum visit in the first 3 weeks					
No	86/151	57	(ref.)	75	(ref.)
Yes	67/102	66	1.2 (0.9–1.4)	82	1.1 (0.9–1.3)

Comprehensive services were defined where all the following domains were documented at any time across the 3 months following birth: (1) sexuality, contraception, and birth spacing (counseling and/or contraceptive provision); (2) infant feeding (documented infant feeding information and/or support from a provider or lactation consultant), (3) chronic disease management (among those with a chronic health issue at the new prenatal visit: hypertensive disorders, diabetes mellitus or a history of gestational diabetes, thyroid disorders, anemia, hyperlipidemia, tobacco use, or mood or anxiety disorder); (4) mood (mental health screen); and (5) physical recovery from birth and/or pregnancy-related health issues (including a blood pressure check in the first 10 days among patients with hypertensive disorders).

^a^ (*n*/*N*): *n* = Number of patients experiencing the outcome divided by *N* = number of patients classified by each level of the characteristic.

^b^ Log-binomial regression was used to estimate RRs.

^c^ Defined as inadequate/intermediate versus adequate/adequate plus using the Kotelchuck index.

^d^ Medical complications of pregnancy include gestational diabetes/diabetes mellitus, hypertensive disorders, anemia, or thyroid disorders.

^e^ Defined for the patient's first prenatal visit provider, because this provider often continued to care for the patient across pregnancy.

^f^ Enabling services included WIC, nutrition, dental, behavioral health, and/or care management.

^g^ Prevalence ratio reported owing to cross-sectional exposure and outcome assessment.

## Discussion

Using novel EHR data from the community health center setting, we characterized quality postpartum care defined through clinical guidelines on the broad structure of the timing and component domains. We found that less than one-third of patients receive quality postpartum care defined by the timeliness of services, whereas nearly two-thirds of patients receive quality postpartum care as defined by comprehensive service measures.

In contrast to the low prevalence of services provided during comprehensive postpartum visits in a national sample,^42^ the majority of patients in our community health center setting received all recommended components of postpartum care. The most prevalent postpartum services included family planning, follow-up regarding recovery from pregnancy and birth, and documentation of infant feeding status.

Fewer patients received more time intensive support for infant feeding from the provider or a lactation consultant, and fewer patients with chronic health issues received targeted follow-up, especially in the case of tobacco use or hypertensive disorders requiring an early postpartum blood pressure check. Among patients who received comprehensive services, most also engaged in timely care; however, the low proportion of patients engaging in timely care overall may be a result of the recent shift in clinical guidance recommending early and ongoing postpartum care beyond a single visit at 6 weeks.

Timely postpartum care and comprehensive postpartum services are two proxy definitions of quality postpartum care for which different factors appear to be predictive. In this exploratory analysis, timely postpartum care was associated with several patient characteristics, including adequate prenatal care use, documentation of a mood or anxiety disorder during pregnancy, and exclusive breastfeeding at the first postpartum visit; one provider characteristic, receiving prenatal care from a family nurse practitioner; and two health center characteristics, early appointment scheduling and receiving enabling services at the first postpartum visit (Table 3).

Adequate prenatal care use has been found to predict postpartum visit attendance across numerous studies.^22,43–49^ Having a mental health disorder has been previously found to correlate with higher postpartum visit attendance where enhanced mental health services are available^50,51^; having a prenatal mood or anxiety disorder may similarly increase timely postpartum care engagement in the community health center setting where enabling services are typically co-located with primary care. Alternately, documentation of a mental health disorder may reflect provider-level engagement, serving as a proxy for the provider's familiarity with the patient and their health history, which is a known predictor of increased health care use following birth.^52^

We found that patients cared for by family nurse practitioners more frequently engaged in timely postpartum care. Nurse providers may be more likely to encourage timely postpartum follow-up or may indirectly increase timely follow-up by spending more time with their patients and establishing strong rapport. A recent survey of a national sample of postpartum care providers found that nurse-midwives and family medicine physicians spend significantly more time with postpartum patients than obstetrician/gynecologist physicians, with nurse-midwives reporting the most time spent with patients.^53^ Future qualitative research should explore patient and provider perspectives on potential mechanisms underlying the association between provider type and timely postpartum care.

We did not observe an association between the racial/ethnic or language concordance of the patient and provider and timely postpartum care. This concordance may be less salient where the majority of providers speak the patient's preferred language. However, previous research has found that perceived discrimination during intrapartum care^54^ and trouble understanding the health care provider^55^ are associated with lower postpartum visit attendance. Future research in community health center communities can complement EHR analyses with patient perspectives of providers' cultural and linguistic competency to further investigate patient–provider relational predictors of quality postpartum care.

In addition, given the significant proportion of Latina and Burmese mothers in our patient population, future qualitative research can explore issues related to acculturation and the effects of the sociopolitical environment that may impact access to quality care for immigrant families served by community health centers.^56,57^

Whereas receipt of enabling services in the prenatal period was not associated with timely postpartum care, postpartum access to these nutrition, infant feeding, and behavioral health services appears to be associated with timely postpartum care, possibly owing to the intersecting maternal and infant health issues that can arise in the early postpartum.^13^ Receipt of enabling services was measured in the early postpartum and may therefore reflect characteristics of patients who would be more likely to return for timely postpartum care, such as parity, insurance status, or maternal age. However, our sample was restricted to a population of mostly low-income and uninsured families for whom socioeconomic characteristics were relatively homogenous and not associated with either quality postpartum care outcome; therefore, these characteristics are not likely to explain the association between receiving enabling services and timely postpartum care.

Whereas data on the timing of scheduling for the postpartum visit were not available for the entire sample, having a postpartum visit scheduled in the first week after delivery was associated with a higher likelihood of timely postpartum care. We do not know whether these missing data result from a lack of early scheduling or a lack of documentation alone; however, other studies have found that postpartum visit appointment reminders^55,58,59^ and access to patient navigators who schedule postpartum visits during the intrapartum hospitalization^51^ are associated with increased postpartum visit attendance.

In settings where perinatal health services are co-located with pediatric and adult primary care, offering dyadic services such as interconception care at regular well child visits may improve maternal risk assessment and offer more frequent opportunities for health care engagement across the postpartum period.^60–62^ Future research can experiment with the timing of visit scheduling to test the effect of early timing on measures of quality postpartum care.

The only correlate of quality postpartum care defined as receipt of comprehensive services was the number of postpartum visits attended across the first 3 months after delivery. This finding reinforces the importance of providing ongoing postpartum care across numerous patient/provider contacts instead of a single postpartum visit at 6 weeks to meet the complex health needs of new parents.^18^ More frequent postpartum visits facilitate multiple opportunities for providers to address intersecting health issues as they arise over time.

Although attending an early 2-week postpartum visit is associated with improved breastfeeding support^63^ and contraception initiation^64^ in other settings, attending an early visit was not associated with receipt of comprehensive postpartum services in this study. The high prevalence of receiving comprehensive postpartum services in our cohort may have contributed to the lack of significant associations with other variables.

Documentation of comprehensive services may have been high in our sample because we conducted comprehensive medical record reviews for data abstraction rather than utilizing administrative or claims data.^65^ Our data abstractors were medical scribes working in the community health center setting who were familiar with the EHR, provider notes, visit flow, and where to identify relevant data. By abstracting data across all visits across pregnancy through 3 months postpartum, we were also able to identify detailed descriptions of the services provided across multiple postpartum visits, which are not available when relying on codes for billing or diagnosis.

Our data abstraction involved mostly free text review to identify and abstract exposure and outcome data, and the availability of variables of interest may have been limited by inconsistent provider documentation. EHR data are not collected for research purposes and often lacks consistent but important patient demographic details such as immigration status, length of time living in the United States, education level, or employment status. We surveyed health care providers for their race/ethnicity and spoken language ability to complement some missing data with potentially informative provider characteristics.

The diverse site characteristics and provider teams of the three sites in our sample facilitated the use of a single EHR to generate findings that may be relevant across different community health center contexts. However, more integrated systems are needed to improve care coordination, document services received outside the community health center, and track referrals for appropriate follow-up care. We were able to confirm receipt of enabling services within the community health center network, such as behavioral health, dental, or nutrition services, but we were not able to link the EHR to external software and relied on provider notes to document receipt of additional services, such as WIC or lactation support.

We were also unable to link the EHR data from the postpartum parent with their infant and are therefore missing any infant feeding or mental health information that may have been documented at well child visits. In the early postpartum period, maternal and infant health behaviors and outcomes are especially interconnected and characterizing the quality of postpartum care requires adapting postpartum visits and EHR documentation to support dyadic health care.

Data abstraction from the EHR of a network of community health centers allowed us to capture patient encounters from postpartum people often missing from research using hospital system or claims data owing to high levels of postpartum insurance loss.^21,66^ We used a causal modeling approach to test associations between exploratory patient, provider, and health center characteristics and outcomes of interest, controlling for all measured confounders that could distort these estimates (Supplementary Table S1). Our estimates may be biased by unmeasured confounding and a lack of clear temporality between exploratory variables and postpartum care outcomes.

Future research can build upon our findings to test these suggested associations between patient, provider, and health center characteristics and quality postpartum care outcomes, especially where factors such as early visit scheduling or the availability of enabling services are amenable to intervention. Finally, we defined quality postpartum care informed by guidelines developed through clinician consensus on the broad structure of postpartum care timing and component domains.^18^

Our findings suggest that different exposures may correlate with quality postpartum care differently depending on how quality care is defined. Future research should engage patients, health care providers, and other stakeholders from marginalized communities to define quality postpartum care measures and identify community-centered practices and policies to improve access to this quality care.

## Conclusions

In our community health center setting, we found a high prevalence of quality postpartum care as defined by comprehensive service measures, but a lower prevalence of timely postpartum care. Our findings suggest that prenatal factors such as adequate care use and an engaged patient–provider relationship, and postpartum factors such as early appointment scheduling, exclusive breastfeeding status, and use of enabling services may increase timely postpartum care. Future research should engage community health center stakeholders to further explore these preliminary findings to translate clinical guidelines for settings serving the most marginalized populations. Identifying best practices from these settings can inform postpartum care strategies to improve maternal health equity.

## Supplementary Material

Supplemental data

Supplemental data

Supplemental data

## Abbreviations Used

ACOG: American College of Obstetricians and Gynecologists
CI: confidence interval
EHR: electronic health record
REDCap: Research Electronic Data Capture
RR: risk ratio
WIC: women, infants, and children
