# Supplementary material for: Correlates of Receiving Guideline-Concordant Postpartum Health Services in the Community Health Center Setting
Source: Womens Health Rep (New Rochelle). 2022 Feb 7;3(1):180–93. doi: 10.1089/whr.2021.0084 (PMC8896220; doi:10.1089/whr.2021.0084)
Supplement: Supplemental data [file Suppl_TableS1.docx]

| **Supplemental Table 1. Variables included in each adjusted risk or prevalence ratio model** | |
| --- | --- |
| **Variable of interest** | **Adjusted for…^a^** |
| Adequacy of prenatal care | Insurance status, parity, maternal age, chronic, health issue, medical issue during pregnancy, preferred language, racial/ethnic concordace of patient/provider, type of provider at the first prenatal visit, number of providers seen across pregnancy |
| Medical issue during pregnancy | Chronic health issue, maternal age |
| Mood or anxiety disorder during pregnancy | Maternal age |
| Parity | Maternal age |
| Insurance status | None (restricted to generally low-income population) |
| Maternal age (years) | None |
| Number of providers seen across pregnancy | Insurance status, parity, maternal age, adequacy of prenatal care, chronic health issue, medical issue during pregnancy, preferred language, racial/ethnic concordace of patient/provider, type of provider at the first prenatal visit |
| Type of prenatal provider | Facility |
| Racial/ethnic concordance of patient/provider | Facility |
| Received enabling services in pregnancy | Number of providers seen across pregnancy, type of provider at the first prenatal visit, facility, insurance status, adequacy of prenatal care, medical issue during pregnancy |
| Delivery type | Maternal age, medical issue during pregnancy, chronic health issue at baseline, parity |
| Postpartum visit scheduled in the first week postpartum | Facility |
| Exclusive breastfeeding at first postpartum visit | Maternal age, medical issue during pregnancy, adequacy of prenatal care |
| Received enabling services at first postpartum visit | Receipt of enabling services in pregnancy, facility |
| Number of postpartum visits in the first three months^b^ | Insurance status, parity, maternal age, adequacy of prenatal care, medical issue during pregnancy, preferred language, racial/ethnic concordace of patient/provider, type of provider at the first prenatal visit, number of providers seen across pregnancy |
| Postpartum visit in the first three weeks^b^ | Insurance status, parity, maternal age, adequacy of prenatal care, medical issue during pregnancy, preferred language, racial/ethnic concordace of patient/provider, type of provider at the first prenatal visit, number of providers seen across pregnancy |

^a^ These final adjustment sets were informed by DAG analysis and consideration of collinearity in our data to further reduce any models were control variables were collinear.

^b^ Considered for comprehensive services outcome only.
