# Supplementary figures and images for: Correlates of Receiving Guideline-Concordant Postpartum Health Services in the Community Health Center Setting
Source: Womens Health Rep (New Rochelle). 2022 Feb 7;3(1):180–93. doi: 10.1089/whr.2021.0084 (PMC8896220; doi:10.1089/whr.2021.0084)

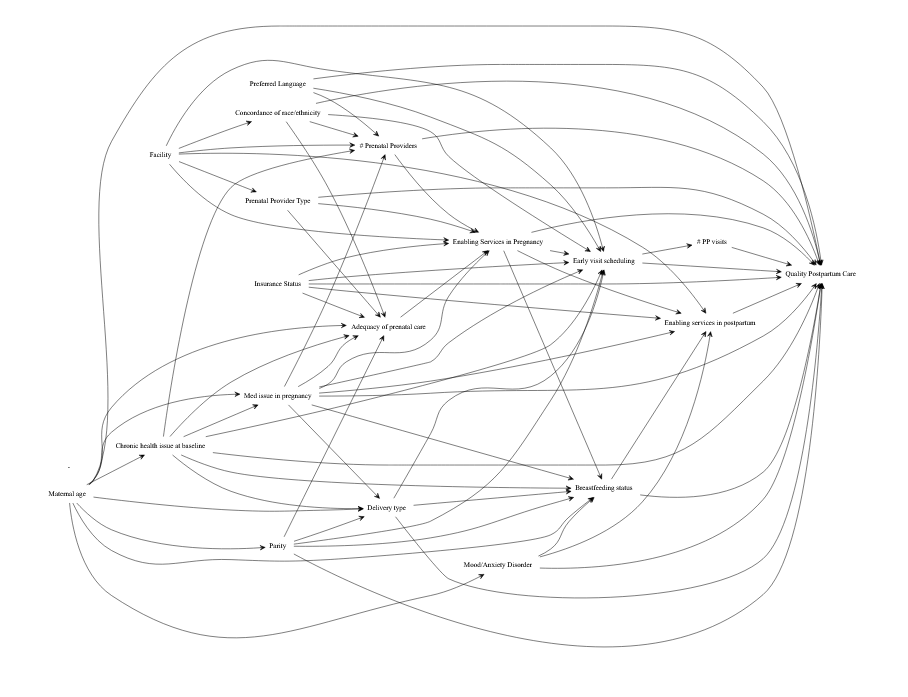

Supplement: Supplemental data [file Suppl_FigS1.PNG]
