# Supplementary material for: Correlates of Receiving Guideline-Concordant Postpartum Health Services in the Community Health Center Setting
Source: Womens Health Rep (New Rochelle). 2022 Feb 7;3(1):180–93. doi: 10.1089/whr.2021.0084 (PMC8896220; doi:10.1089/whr.2021.0084)
Supplement: Supplemental data [file Suppl_TableS2.docx]

**Supplemental Table 2. Cross Tabulation of Two Quality Postpartum Care Outcomes**

|  | **Comprehensive postpartum services** | | |
| --- | --- | --- | --- |
| **Timely care** | **No** | **Yes** | **Total** |
| **No** | 80 (31.6) | 95 (37.6) | 175 (69.2) |
| **Yes** | 20 (7.9) | 58 (22.9) | 78 (30.8) |
| **Total** | 100 (39.5) | 153 (60.5) | 253 |
